# Hydroponic Ginseng ROOT Mediated with CMC Polymer-Coated Zinc Oxide Nanoparticles for Cellular Apoptosis via Downregulation of *BCL-2* Gene Expression in A549 Lung Cancer Cell Line

**DOI:** 10.3390/molecules28020906

**Published:** 2023-01-16

**Authors:** Yinping Jin, Esrat Jahan Rupa, Jinnatun Nahar, Li Ling, Aditi Mitra Puja, Reshmi Akter, Deok Chun Yang, Se Chan Kang, Hao Zhang

**Affiliations:** 1 Institute of Special Wild Economic Animals and Plants, Chinese Academy of Agricultural Sciences, Changchun 130112, China; 2 Department of Biotechnology, College of Life Science, Kyung Hee University, Yongin-si 17104, Gyeonggi-do, Republic of Korea

**Keywords:** lung cancer, ZnO NPs, apoptosis, hydroponic ginseng root

## Abstract

The unique and tailorable physicochemical features of zinc oxide nanoparticles (ZnO-NPs) synthesized from green sources make them attractive for use in cancer treatment. Hydroponic-cultured ginseng-root-synthesized ZnO-NPs (HGRCm-ZnO NPs) were coated with O-carboxymethyl chitosan (CMC) polymer, which stabilized and enhanced the biological efficacy of the nanoparticles. Nanoparticles were characterized by X-ray diffraction (XRD), UV-Vis spectroscopy, transmission electron microscopy (TEM), Fourier-transform infrared spectroscopy (FT-IR), and energy-dispersive X-ray spectroscopy (EDS). The flower-shaped nanoparticles were crystalline in nature with a particle size of 28 nm. To evaluate if these NPs had anti-lung cancer activity, analysis was performed on a human lung carcinoma cell line (A549). HGRCm-ZnO nanoparticles showed less toxicity to normal keratinocytes (HaCaTs), at concentrations up to 20 µg/mL, than A549 cancer cells. Additionally, these NPs showed dose-dependent colony formation and cell migration inhibition ability, which makes them more promising for lung cancer treatment. Additionally, Hoechst and propidium iodide dye staining also confirmed that the NP formulation had apoptotic activity in cancer cells. Further, to evaluate the mechanism of cancer cell death via checking the gene expression, HGRCm ZnO NPs upregulated the *BAX* and *Caspase 3* and *9* expression levels but downregulated *Bcl-2* expression, indicating that the nanoformulation induced mitochondrial-mediated apoptosis. Moreover, these preliminary results suggest that HGRCm ZnO NPs can be a potential candidate for future lung cancer treatment.

## 1. Introduction

Cancer is an alarming global disease with a high mortality rate, with an estimated 19.3 million new cancer cases and almost 10.0 million cancer deaths in 2020 according to Global Cancer Statistics [1]. According to estimates from the World Health Organization (WHO) in 2019, cancer is the first leading cause of death before age 70. Lung cancer remains the leading cause of cancer death, with an estimated 1.8 million deaths (18%) [2]. Non-small cell lung cancer (NSCLC) is responsible for lung malignancy for about 85 % of all lung cancers with lower therapeutic activity [3]. Cancer metastasis is the main challenge in the lung cancer management system, even though clinical treatment and management systems of cancer are developing with different conventional therapies [4,5]. Several cancer treatment categories, including radiotherapy, chemotherapy, immunotherapy, surgical resection, and targeted therapy, are widely used. Initially, chemotherapy drug treatment exposes positive feedback and enhances the quality of the patient’s life. However, chemotherapeutic drugs by their very nature are toxic, with numerous side-effects, and drug resistance can evolve [6]. Although the combination of several medicines increases the survival rate of cancer patients, the overall result is still unsatisfactory for the treatment of cancer. Nanostructured materials are currently of great interest in the cancer world as potential treatment options to diagnose, treat, and prevent cancer [7]. Nano-constructed drug delivery systems have significantly improved drug delivery to the target site compared with traditional administration processes. Moreover, nano-based drug delivery systems show effective targeting, delayed release, and increased bioavailability and are less toxic than traditional delivery systems [8]. Physical and chemical methods are used to synthesize nanoparticles (NPs), and some researchers have focused on developing eco-friendly methods for synthesizing nanoscale materials [9]. Green synthesis processes are considered simple, inexpensive, and non-hazardous [10,11].

Different metal oxide nanoparticles, such as Zn, Ag, Mg, Cu, Pt, and Al NPs, have been efficiently produced from phytoextracts using green synthesis methods [12,13]. ZnO nanoparticles are of great interest in the anticancer field because of their high biocompatibility, low toxicity, and cost-effectiveness [14,15]. Zinc oxide (ZnO) is an inorganic agent that is a generally recognized as safe (GRAS) compound by the United States Food and Drug Administration (US-FDA) [16]. ZnO can be prepared at the nanoscale level using various methods such as the sol-gel method [17], co-precipitation method [18,19], laser ablation [20], or hydrothermal synthesis method [21]. However, here, we chose to use a co-precipitation synthesis method for ZnO-NP formation by coating the ZnO-NPs with a biodegradable polymer, o-carboxymethyl chitosan (CMC) [22]. Carbohydrate polymers, also known as polysaccharides, are abundant in nature. These molecules comprise covalently linked monosaccharide molecules. A well-known polysaccharide is chitosan, a linear polysaccharide produced by the deacetylation of chitin; this polymer displays remarkable mucoadhesion, biocompatibility, and chemical versatility [23]. Chitosan and chitosan-based nanocomposites have been shown to be nontoxic, biocompatible, and biodegradable, with antibacterial [24], anticancer [25,26], and immune-enhancing effects [27]. These characteristics have made chitosan a suitable choice for various biomedical applications. Additionally, zinc oxide NPs have potential efficacies in the medicinal sector, for example, anticancer [28], antioxidant [29], antimicrobial [30], antidiabetic [31], and antiviral [32] activities. A previous study reported that the green synthesis of ZnO-NPs containing *Dendropanax morbifera* extract [33], Ginsenoside Rh2 [34], or *Echinacea purpurea* callus extract [35] exhibited significant anticancer activity.

*Panax ginseng* Meyer has been widely used in Asian traditional medicine to treat a variety of diseases for thousands of years [36]. Ginseng saponins, also known as ginsenosides, have significant pharmacological activities. More than 289 bioactive components have been reported from 11 *Panax* species [37]. Mostly, 5–6-year-old ginseng is used in the commercial sector, but the Rural Development Administration of Korea developed a new, shorter-duration (~120 days) hydroponic technology for ginseng cultivation [38]. In hydroponic systems, plants are grown with their roots in nutrient-enriched water instead of soil and without any crop-protective agents [39]. When using the hydroponic method, the growth environment, including light intensity, temperature, and moisture content, can be tightly controlled. Therefore, the short-term cultivation of a hydroponic culture results in a higher content of ginsenosides in the roots and leaves [40]. Hydroponic-cultured ginseng saponins have antioxidant [39], anti-inflammatory [41], hepatoprotective [42], and immunomodulatory activities [43], but there is no scientific evidence that hydroponic cultured fresh ginseng root is pharmacologically efficacious against lung cancer. 

In this study, we synthesized nano-based ZnO particles using 120-day-aged hydroponically cultured fresh ginseng root via a green synthesis method [33]. Due to the biocompatibility of these ZnO NPs with normal human cells and their high dissolution rate, the intracellular ZnO NPs showed cancer cell cytotoxicity at a slightly acidic pH [44] and were found to be safe anticancer agents. To protect and enhance the biological efficacy of fresh hydroponic ginseng root, this study focused on synthesized ZnONPs coated with a (CMC) polymer as a green-synthesized nanodrug for lung cancer treatment.

## 2. Results

### 2.1. Characterization of HGRCmZnO NPs

**Figure 1:**
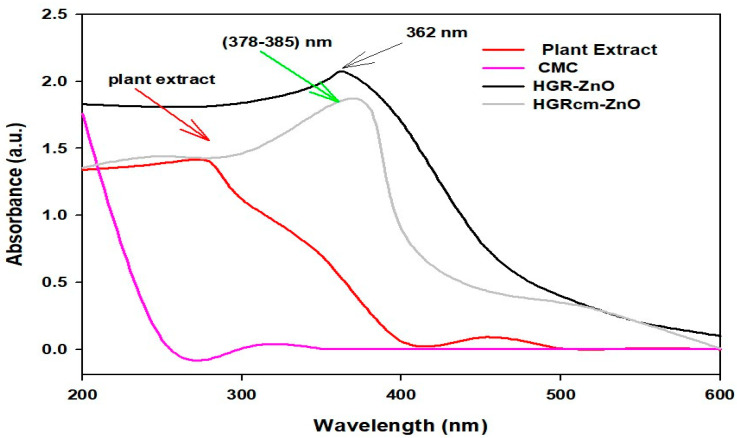
UV-visible spectroscopy confirmation of the formation of hydroponic ginseng root CMC-coated ZnO NPs (HGRCm-ZnO NPs).

UV-Visible spectra of ZnO-NPs synthesized using a hydroponic ginseng root extract, those coated with CMC (HGRCm-ZnO NPs), plant extract alone, and CMC alone are shown in Figure 1. The successful formation of ZnO-NPs was indicated by the presence of a peak at 362 nm. Moreover, the plant extract showed a broader peak at 280 nm, representing the polyphenolic compound mainly responsible for nanoparticle formation and neutralizing the metal ion [44]. CMC-coated nanoparticles were characterized by peaks at 378–385 nm due to the presence of CMC strongly bonded with amine groups and metal ions [45]. Additionally, metal nanoparticles are known to combine with polyphenols, including anthocyanin, a highly reactive substance; therefore, we examined if an anthocyanin peak was present [33]. Phytochemicals (phenol, flavonoid, and rutin) present in ginseng root function as reducing agents, converting metal Zn^+2^ to Zn^0^, which also represent stable nanoparticles. The plant extract contained the polyphenolic group and other phytochemicals that showed an absorption zone at 280 nm, whereas ZnO NPs had a sharp peak at 362 nm. However, the plant extract alone had no sharp peak on the metal nanoparticle formation area. Zinc nitrate salt is converted to zinc ions and further processed to stabilized zinc metal due to reduction by phytochemicals in ginseng root. The CMC polymer containing amines bonded strongly with metal ions in the ZnO NPs and acted as a coating agent. 

**Figure 2:**
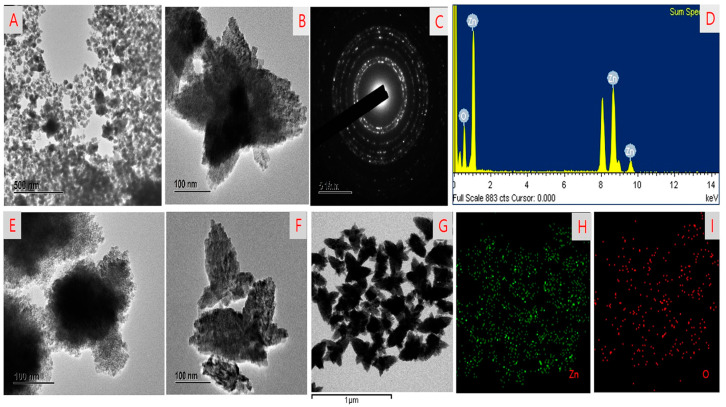
FE-TEM images showing the morphology of (**A**,**E**) HGR-ZnO NPs without CMC; (**B**,**F**,**G**) ZnO NPs with CMC; (**C**) SAED results; (**D**) EDX results; and (**H**,**I**) elemental mapping of HGRCm-ZnO NPs.

The morphology of HGRCm-ZnO NPs in different nanoscale ranges was examined by FE-TEM. Nanoparticles without the CMC polymer had an aggregated structure and did not appear to be stabilized at a higher magnification (40 nm) (Figure 2A,E). Interestingly, CMC-coated nanoparticles had a flower-like shape, and higher magnification images confirmed a 5-6-petal-like structure (Figure 2B,F,G). A previous study reported that flower-shaped NPs had better anticancer efficiency in the endothelial cell than normal-shaped NPs [46]. In addition, SAED patterns were used to examine the crystallinity of nanoparticles (Figure 2C). Moreover, EDX confirmed the presence of Zn and O in the ZnO nanoparticles (Figure 2D). Elemental mapping highlighted the zone of oxygen and zinc in the nanoparticles (Figure 2H,I).

**Figure 3:**
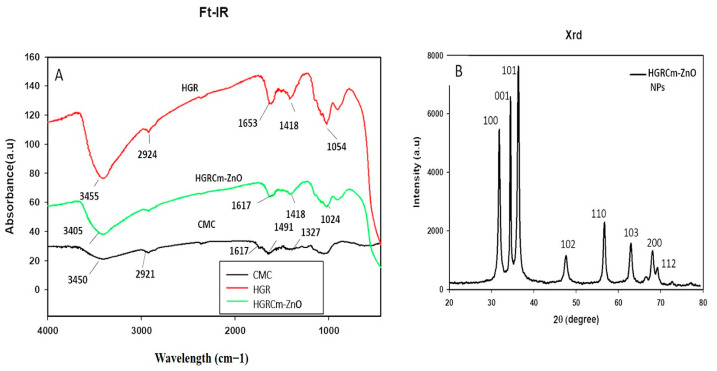
(**A**) Fourier transforms infrared analysis (FT-IR) for CMC (Black), hydroponic ginseng root (Red) and HGRCm-ZnO NPs (Green) and (**B**) XRD analysis of hydroponic ginseng root CMC-coated ZnO NPs (HGRCm-ZnO NPs (Black).

FT-IR analysis: The phytochemicals present in the ZnO NPs were identified using FT-IR analysis. The characteristic peaks of HGRCm-ZnO NPs, CMC, and ginseng root alone are highlighted in the FT-IR analysis in Figure 3A. Nanoparticles exhibited a broad peak in the region of 3000–3500 cm^−1^. We attributed the peak at 3455 cm^−1^ to (-OH) and N-H amide bonds contributed by secondary amines and polyphenols present in the ginseng extract and CMC polymer. The peak at 1617 cm^−1^ was attributed to N-H and phenolic bonds from CMC and the ginseng extract. Previous studies reported that the peak at 1327 cm^−1^ is due to C-H ether bonds from the polymer [47,48].

An XRD graph showing the crystalline nature of the nanoformulation is presented in Figure 3B. The crystallite size of the nanoparticles was calculated using the full width at half-maximum (FWHM) value of the nanoformulation and was 28 nm (Table S1). The peak intensity and FWHM values were computed using the 2θ range of 20–80°. The nature of the nanoparticles was indexed according to the miller index using *h*, *l*, and *k* at lattice planes (100), (001), and (101), respectively. Figure 3 shows that the NPs had a hexagonal wurtzite structure that was an identical match to the standard values of ZnO nanocrystals (JCPDS NO. 36−145) [22]. Moreover, the absence of an extra peak in the nanoformulation confirmed its purity. 

### 2.2. Cell Viability Analysis

**Figure 4:**
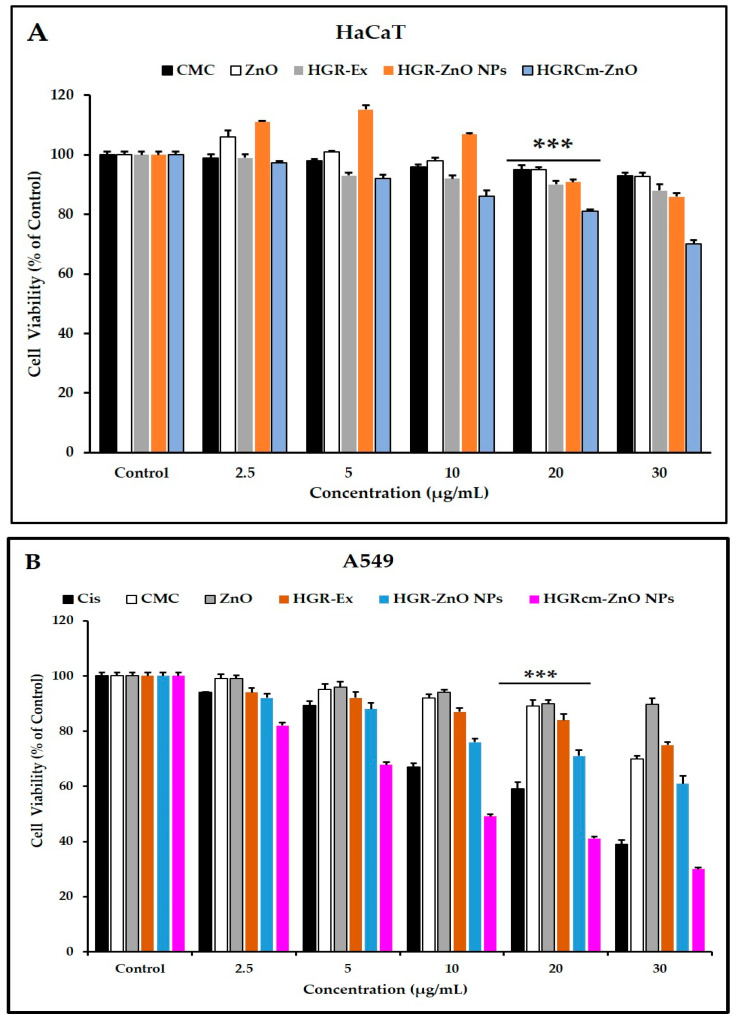
Cell viability of HGRCm-ZnO NPs, CMC, root extract, and cisplatin based on the MTT assay. (**A**) HaCaT cells; (**B**) A549 cells. Each bar represents the mean ± SE of duplicate samples from three independent experiments (*** *p* < 0.001 using Student’s *t*-test compared to the control.).

The MTT assay was used to assess the anticancer activity of different concentrations of HGRCm-ZnO NPs (control, 2.5, 5, 10, 20, and 30 µg/mL). Commercial cisplatin was used as the positive control (Figure 4B), and non-treated cells were used as a control. HGRCm-ZnO NPs significantly inhibited the growth of cancer cells at 20 µg/mL, with a cell viability of only 45% compared with ZnO salt, extract, ZnO NPs (without CMC), or CMC alone (Figure 4B). HaCaT cells treated with HGRCm ZnO had a cell viability of 75% (Figure 4A). Accordingly, the results of IC50 values also showed that HGRCm-ZnO NPs (IC50 =15.74 μg/mL) was more potent against A459 cells than the normal cells (IC50 = 52.56 μg/mL) (Table S2). 

Morphological alterations of A459 cells were observed after a 24 h treatment with HGRCm-ZnO NPs. In Figure S1, the morphological alternation of A549 cells treated with HGRCm-ZnO NPs at a concentration of 20 µg/mL versus untreated cells demonstrates that the untreated cells had a more significant phenotype in the cell morphology. In contrast, the treated cell shrinkage had reduced size and was highly detached from the colony. HGRCm-ZnO NPs showed significantly higher cytotoxic efficacy than ZnO NPs (Figure S1), and this is likely due to the combined effects of the ginseng extract and CMC polymer. 

The greater cancer cell toxicity of HGRCm-ZnO NPs may be due to the high concentration of zinc bound to different proteins. In contrast, the amount of free Zn^2+^ ions are still very low and is tightly regulated by homeostatic mechanisms [49,50]. Further, ZnO nanoparticles have high dissolution capacity in acidic media, such as intracellular lysosomal compartments, and hydrated zinc ions combine with intact ZnO nanoparticles, which is thought to cause mitochondrial damage and disrupt cellular zinc homeostasis, ultimately resulting in cell death [51,52]. ZnO NPs have the ability to rapidly cross cell membranes to damage mitochondria and particular DNA sequences, dramatically inhibiting the development of tumors at specific sites [50]. Overall, HGRCm-ZnO NPs significantly slowed cancer cell proliferation due to the combined effects of samples.

### 2.3. Colony Formation

**Figure 5:**
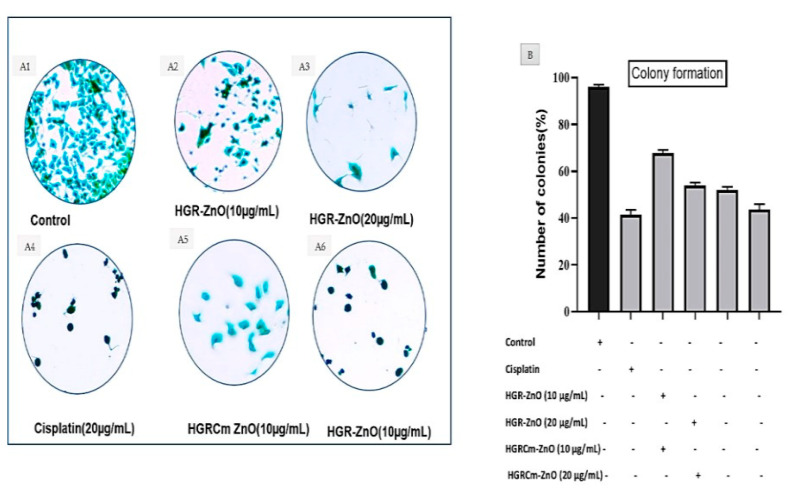
The colony formation assays. (**A**) The representative image of the colony formation after being stained with crystal violet of HGRCm-ZnO NPs at 10 and 20 µg/mL. Cisplatin was used as a positive control. (**B**) The number of colonies was counted using Image J analysis software, and the results are presented in graph.

A clonogenic test, also known as a colony-forming assay, is an in vitro quantitative approach to assess a single cell’s capacity to increase into a large colony by clonal expansion. As shown in Figure 5, the cell morphology and colony development of A549 cells were assessed by microscopic examination. Control group cells (Figure 5A1) contained more A549 cell colonies than cells treated with HGR-ZnO NPs (Figure 5A2, A3) and HGRCm-ZnO NPs at 10 and 20 μg/mL, respectively (Figure 5A5, A6). Notably, at the same concentrations, HGRCm-ZnO NPs decreased the number of colonies significantly more than HGR-ZnO NPs. Additionally, 20 μg/mL HGRCm-ZnO NPs inhibited colony development significantly compared with the control group. Our findings suggest that the anticancer activity of HGRCm-ZnO NPs involves the inhibition of colony formation without significant toxic effects on normal cells (see Figure 5B).

### 2.4. HGRCm-ZnO NPs Inhibit Migration of Cancer Cells

**Figure 6:**
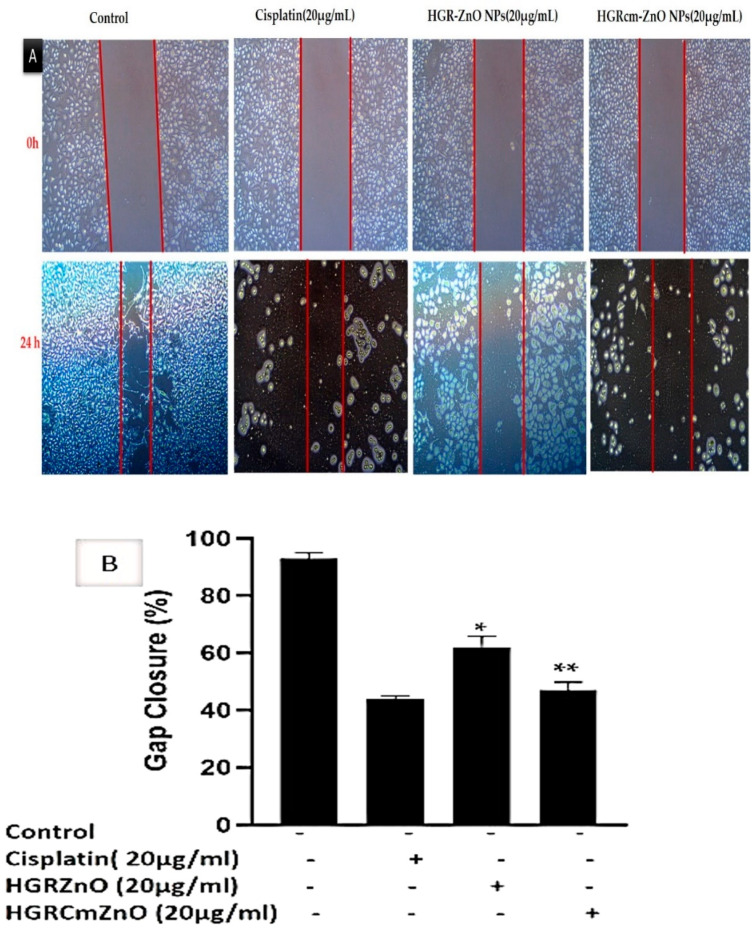
(**A**) The cell-free area of the scratched region was measured with ImageJ software. The extent of cell migration is presented as the percentage of scratch (wound) closure observed 24 h after treatment compared to control values (**B**). Controls indicate untreated cells. Values are expressed as mean ± standard deviation, and * *p* < 0.01; ** *p* < 0.01 indicates significant differences from control groups. The scale bar indicates 10× magnification.

Overall, 90% of cancer deaths are due to cancer metastasis; therefore, the prevention of cancer metastasis is a major goal of cancer treatment [53]. Cell migration as cancer progresses determines the ability of tumor cells to escape from primary tumors and invade nearby tissues to become metastases. A scratch migration assay was performed to determine if HGRCm-ZnO NPs affected the migration of A549 cells. A549 lung cancer cells were treated with HGRCm-ZnO NPs (20 µg/mL), and cell migration (%) was evaluated using a wound closure assay before and after the treatment of cells (Figure 6A,B). Cells treated with HGRCm-ZnO NPs showed strongly inhibited migration after 24 h. The percentage of A549 lung cancer cells that migrated toward the scratch area was 92.0 ± 0.8% for untreated control cells, 60.0 ± 0.2% for those treated with HGR-ZnO NPs (without CMC), 45.0 ± 0.4% for those treated with HGRCm-ZnO NPs, and 39.0 ± 0.6% for those treated with the positive control drug cisplatin (Figure 6B). These results indicate that HGRCm-ZnO NPs have the ability to inhibit cell migration and cancer cell metastasis. After 24 hours, practically all gaps between cell layers in the control group were covered by migrating cells. Additionally, cancer cells treated with HGRCm-ZnO NPs displayed better migratory inhibition ability than the control group and ZnO NPs (without CMC). In contrast, due to the decreased migratory capabilities of HGRCm-ZnO NP-treated cells, scraped areas were still apparent. The capacity of HGRCm-ZnO NPs to suppress growth may indicate that it is a potential anti-lung cancer agent.

### 2.5. Detection of HGRCm-ZnO NP-Induced Apoptosis by Hoechst-33342/PI Dye Staining

**Figure 7:**
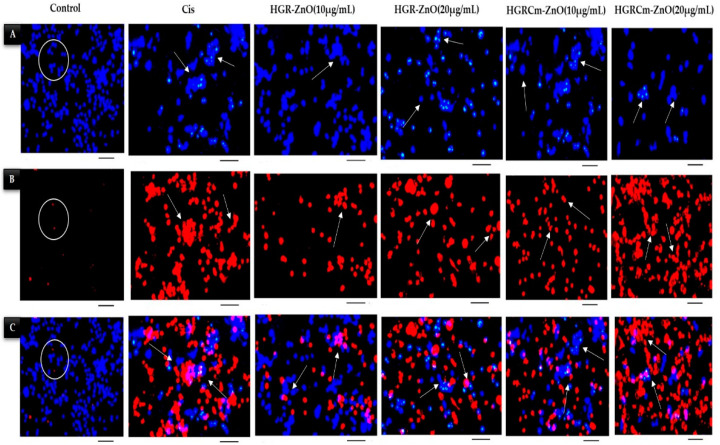
Hoechst and PI staining of HGRCm-ZnO NPs and detection of cellular apoptosis via cell disruption and breakage of the cell wall, as indicated with arrowheads. (**A**) Hoechst staining (light-blue live cells and dark-blue apoptotic cells), (**B**) PI staining (dark-red dead cells), and (**C**) merged images. Original scale bar 20×.

Apoptosis is a vital physiological process important for homeostasis and the maintenance of differentiated cells [54]. Therefore, the activation of apoptosis in cancer cells is one of the molecular bases for anticancer therapeutic approaches. In addition, pyknosis and cell shrinkage are observed early in the process of apoptosis. Organelle condensation and cytoplasmic density cause cell shrinkage, and chromatin condensation (pyknosis) is the most important aspect of early apoptosis [55]. In this study, we used a Hoechst/PI double-staining assay to detect apoptosis after 24 h treatment of cells with HGRCm-ZnO NPs. Live cell nuclei were stained light-blue (Hoechst dye), while apoptotic cells were stained dark-blue. Dead cells were stained with PI dye (dark-red), as indicated by the arrowheads in Figure 7. The control group cells were stained blue, with no red cells apparent. When cells were treated with 10 µg/mL HGRCm-ZnO NPs, the cell number started to decrease. At 20 µg/mL HGRCm-ZnO NPs, a large number of dead cells were found after PI staining (Figure 7B). Massive numbers of necrotic cells were found in increased concentration under high magnification, and changes in nuclei and cell morphology were observed in cells treated with HGRCm-ZnO NPs, as can be seen in Figure 7 (marge section). The HGRCm-ZnO NPs and HGR-ZnO NPs (without CMC) and commercial drug cisplatin effect were also investigated for comparison. Cells treated with 20 µg/mL HGRCm-ZnO NPs showed membrane instability and cytoskeletal disturbances with the highest number of dead cells, comparable to that observed with cisplatin. Although a number of molecular markers are available to investigate the mechanism of cell death, morphological criteria remain the standard for defining the mode of cell death [56]. The fluorescent substance propidium iodide (PI) binds primarily between DNA nucleotides. So, nuclear changes were observed using PI staining. We observed nuclei with an apoptotic morphology, as characterized by bright-red condensed nuclei (intact or fragmented). Control cells had round, intact, red nuclei, as PI dye was unable to enter the cell through the cell membrane (Figure 7B). These results indicate that the cell membrane ruptured after treatment with HGRCm-ZnO NPs, allowing the dye to enter the injured cell and stain the nucleus, thereby indicating that apoptosis was the mode of cell death.

### 2.6. HGRCm-ZnO NPs Induce Apoptosis by Regulating Apoptotic Gene Expression

A549 lung cancer cells were treated with different concentrations of HGRCm-ZnO NPs, and mRNA expression was quantified. There was a steady decrease in *Bcl-2* transcript expression at 10 µg/mL HGRCm-ZnO NPs (0.6-fold), while a 0.4-fold decrease in expression was observed at 20 µg/mL HGRCm-ZnO NPs. *BAX* mRNA expression was increased relative to control levels after treatment with 10 µg/mL HGRCm-ZnO NPs (1.2 fold) and further increased with 20 µg/mL HGRCm-ZnO NPs (1.6-fold).

**Figure 8:**
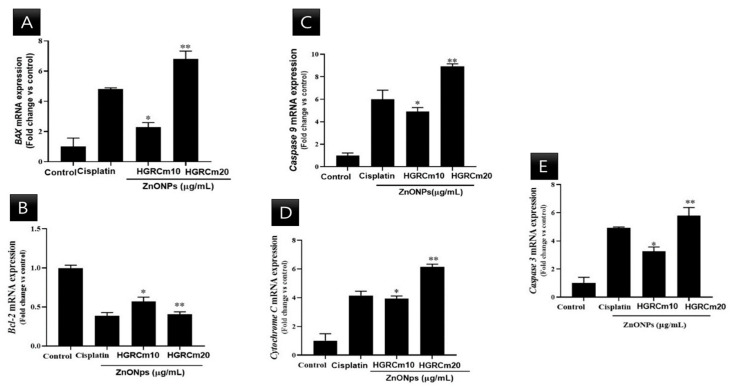
Effects of HGRCm-ZnO NPs on mRNA expression levels of apoptosis-related genes in A549 cells. Here, A549 cells were treated with HGRCm-ZnO NPs at 10 and 20 μg/mL for 24 h. Total RNA was then extracted, and transcript expression levels were determined by qPCR analysis using primers targeting (**A**) *BAX*, (**B**) *Bcl-2*, (**C**) *Caspase 9*, (**D**) *Cytochrome C*, and (**E**) *Caspase 3*. Each bar represents the mean ± SE of duplicate samples from three independent experiments. * *p* < 0.01; ** *p* < 0.01 using Student’s *t*-test compared to the non-treated control).

**Figure 9:**
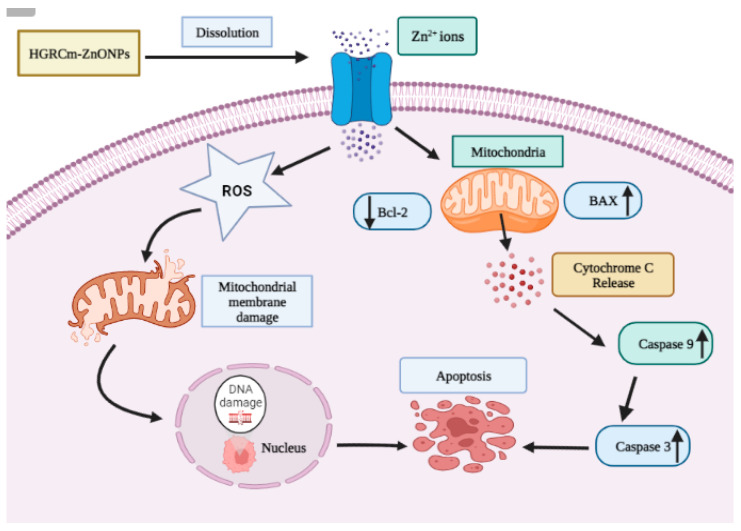
Cellular apoptosis via the intrinsic mitochondrial pathway. The intrinsic pathway is activated by intracellular stimuli such as DNA damage or oxidative stress. Activation of mitochondrial-mediated apoptosis, which is indicated by inhibition of *Bcl-2* and an increase in *BAX,* permits the release of *Cyt c* into the cytoplasm and, ultimately, the upregulation of *Caspase 9/3* genes to activate the intrinsic apoptotic signaling cell death process.

Figure 8 shows that the expression of *Bcl-2* decreased significantly after 20 µg/mL HGRCm-ZnO NP treatment; at the same time, *BAX* (anti-apoptotic) expression gradually increased. ROS, produced by mitochondria, has been identified as an essential molecule in stress signaling in cells. The alteration of the redox status reaction due to increased ROS production makes cells more vulnerable to oxidative stress and apoptosis [57]. There are two major apoptotic signaling routes: the intrinsic and extrinsic pathways. The intrinsic pathway is activated by intracellular stimuli such as DNA damage or oxidative stress. The *Bcl-2* protein family consists of pro and anti-apoptotic proteins that strictly regulate the intrinsic route via the mitochondria. During apoptosis induction, levels of pro-apoptotic *BAX* increase, resulting in the inhibition of the activity of the anti-apoptotic protein *Bcl-2* and mitochondrial outer membrane premiumization (MOMP) [58]. Disruptions in mitochondrial membrane potential allow cytochrome c (Cyt c) to be released into the cytosol. *Caspase 9* and *caspase 3* are activated and cellular apoptosis occurs. *Cyt c* is a critical activator of *caspases 9* and *3* (Figure 9). We found that HGRCm-ZnO NPs inhibited the expression of the anti-apoptosis gene *Bcl-2* by stimulating pro-apoptotic gene expression.

## 3. Materials and Methods

### 3.1. Plant Materials

Hydroponically cultured ginseng root samples were collected from Hanbang Bio Laboratory, Kyung Hee University, South Korea.

### 3.2. Chemicals

Zinc nitrate hexahydrate (>98%) and NaOH (>98%) were supplied by Dae-Jung Chemicals and Metals Co., Ltd. (Pyeontaek, Korea). Absolute alcohol, Tween 80, and olive oil were purchased from Samchun Pure Chemical Co. Ltd. (Gyeonggi-do, Korea). The human keratinocyte cell line (HaCaT) and lung cancer cell line (A549) used in this study were attained from the Korean cell line bank (Seoul, Korea). Cell culture reagents such as RPMI 1640, DMEM, penicillin–streptomycin, and fetal bovine serum (FBS) were obtained from Gen DEPOT Inc. (Barker, TX, USA), Gibson-BRL (Grand Island, NY, USA), and WElGENE Inc. (Daegu, Korea). Dimethyl sulfoxide (DMSO), MTT reagents, and Hoechst-33342 dye were obtained from Sigma-Aldrich (St. Louis, MO, USA). Invitrogen (Carlsbad, CA, USA) provided propidium iodide (PI) dye. Green/ROX QRTPCR Master Mix was obtained from Thermo Scientific (Foster, CA, USA). 

### 3.3. Preparation of HGR Extract

At first, root samples were thoroughly washed with distilled water and all debris was eliminated. Samples were dried under dust-free conditions. Dried roots were then crushed into a fine powder. Five grams of the fine powder was mixed in distilled water (100 mL) in a conical flask and placed into the autoclave machine for 40 min at 100 °C under maximum pressure to extract phytochemicals from the sample. Liquid root extracts were collected using No. 1 filter paper by filtration and then centrifuged at 4500 rpm to remove unwanted components. The supernatant was stored at 4 °C for further tests.

### 3.4. Synthesis of GR-ZnO NPs from the Extract

**Figure 10:**
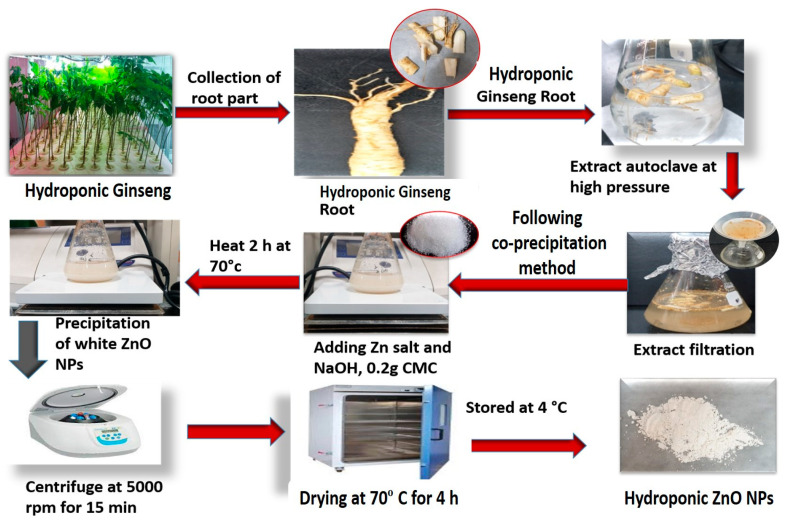
Synthesis of HGRCm-ZnO NPs from hydroponic ginseng root and CMC polymer.

A previously described co-precipitation method was performed to prepare HGR-ZnO NPs with slight modification [59]. In the following process, zinc nitrate and sodium hydroxide were used as oxidizing salt and precipitating precursor, respectively. Metal nitrates are extremely soluble and have an oxidizing capacity and are ideal for the preparation of nanoparticles. Distilled water was used to wash away unreacted salt and phytochemicals from the nanoparticles. At first, ten percent (20 mL) hydroponically cultured ginseng root extract (*w*/*v*) was dissolved in distilled water (80 mL) under stirring, and 0.1 mM of zinc nitrate salt was then added. The solution was stirred continuously (500 rpm) on a hotplate with the temperature maintained at 65 to 70 °C. Then, 0.2 M of NaOH-prepared solution was mixed slowly dropwise into the hot solution over 2 h. The solution was permitted to become cold without stirring and then centrifuged at 8000 rpm for 15 min to eliminate unmixed impurities. After completion of the reaction, the white precipitate that formed was permitted to settle, and the supernatant was removed. The formed GR-ZnO NPs were cleaned with DW three times to remove unreacted substances and dried at 60 °C for 4 h in an oven. During this drying period, the Zn (OH)_2_ in the NPs converted fully into white color ZnO NP powder (Figure 10).

## 4. Characterization

Structural and optical properties of the synthesized ZnO nanoparticles were determined through different diagnostic machines for measuring particle morphology, specific size, and stability.

### 4.1. UV-Vis Spectrophotometry 

The preparation of GR-ZnO NPs was examined using UV-Vis spectroscopy (Ultrospec TM-2100 Pro) operated between 200 and 700 nm.

### 4.2. FT-IR

An FT-IR (PerkinElmer Inc., Waltham, MA, USA) machine was used to investigate the spectra of zinc oxide nanoparticle powder at wavelengths between 4000 and 450 cm^−1^. Spectral properties are presented in plots of transmittance (%) versus wavenumber (cm^−1^).

### 4.3. XRD Analysis

The crystallinity and specific size of GR-ZnO nanoparticles were evaluated via X-ray diffraction (D8 Advance, Bruker, Germany) with a detector voltage of 40 kV, current of 40 mA, and Cu-Kα radiation of 1.54 Å. Properties were recorded in the 2θ range of 20–80° with rapid scanning (6°/min).

### 4.4. FE-TEM Analysis

The structural morphology and particle size of ZnO-NPs were determined using a TEM (200 kV) JEM-2100F (JEOL, Japan). A short-time slight film of a carbon-coated copper grid was prepared to measure the size of the NPs. Extra solution was cleaned with filter paper and then set in a grid box serially.

### 4.5. Cell Culture

A549 human lung cancer cells were grown in RPMI 1640 89% supplemented with 10% FBS and 1% P/S. Normal human keratinocyte (HaCaT) cells were grown in DMEM supplemented with 10% FBS and 1% P/S. Cells were incubated at 5% CO_2_ in a 37 °C incubator and were permitted to adhere and grow prior to treatment.

### 4.6. Cell Viability Assay

The cytotoxicity of hydroponic-cultured ZnO NPs against A549 and HaCaT cell lines was assessed using the MTT assay. Initially, cancer cells and normal cells were plated at a density of 1 × 10^4^ cells/well in a 96-well plate. Cells were then treated with several concentrations of ZnO NPs. After 24 h, cells were treated with 20 µL of 3-(4,5-dimethyl-2-thiazolyl)-2,5-diphenyl tetrazolium bromide solution (MTT; 5 mg/mL, PBS; Life Technologies, Eugene, OR, USA) for 4 h at 37 °C. Then, 100 µL of DMSO was added to every well to dissolve insoluble formazan crystals. Last, a microplate shaker was used to dissolve the formazan crystals for 10 min in the dark. Absorbance at 570 nm was recorded using an ELISA plate reader (Bio-Tek, Instruments, Inc., Winooski, VT, USA).

### 4.7. Colony Formation

A549 lung cancer cells were seeded at a concentration of 1 × 10^3^ in the wells of a 6-well plate, and after 48 h, cells were treated with different concentrations (5, 10) µg/mL of HGRCm-ZnO NPs. Control cells were treated with 0.1% DMSO. After 1 day of incubation, the old cell media were removed, and new media were added for 7 days. Colonies were fixed with glutaraldehyde and stained with crystal violet. Images were captured under a microscope, and colony number was counted and plotted. All data were analyzed using Image J software

### 4.8. Wound-Healing Assay

The cell migration ability of A549 cancer cells was determined using a wound healing assay. A549 lung cancer cells were seeded in 6-well plates at 2 × 10^4^ cells per well and incubated at 37 °C for 24 h. The monolayer was scratched vertically using a 200 µL sterile pipette tip, and detached cells were removed using PBS. Cells were then treated with different concentrations, 10 or 20 µg/mL, of HGRCm-ZnO NPs, and after 72 h of treatment, images were captured using an integrated 5.0 megapixel MC 170 HD camera (Wetzlar, Germany).

### 4.9. Hoechst Staining

A Hoechst-33342 staining kit was used to assess if HGRCm-ZnO NPs induced apoptosis of A549 cancer cells. Cells were placed into a 6-well plate at a density of 1 × 10^4^ cells/well in 2 mL culture media and incubated for 24 h. Then, 4% paraformaldehyde was added for 10 min after washing treated cells with 1× PBS solution (twice). After a 10 min incubation at 37 °C, 10 μg/mL Hoechst dye was added. Stained cells were washed three times in PBS solution, and cell images were captured under a fluorescence microscope (Leica DMLB, Wetzlar, Germany).

### 4.10. PI Staining

Seeded cells were treated with 10 or 20 μg/mL HGRCm-ZnO NPs. After a 24 h treatment period, cells were washed with 1 mL 1× PBS and stained with 500 μL propidium iodide reagent (5 μg/mL) solution at room temperature for 10 min. Cells were observed using a fluorescence microscope (Leica DMLB, Wetzlar, Germany). 

### 4.11. Quantitative Real-Time PCR 

A RNeasy mini kit was used to extract total RNA from treated and non-treated cells after 48 h of treatment. Before extraction, cells were seeded in 25 cm^2^ cell culture plates (NY, USA) and treated with 1, 2.5, 5, 10, 20, or 30 μg/mL of HGRCm-ZnO NPs. For real-time quantitative PCR (qRT-PCR), 500 ng of RNA was reverse-transcribed using oligo (dT) 15 primer (0.2 mM), and cDNA was synthesized using a cDNA kit (Invitrogen, CA) following the manufacturer’s instructions. qPCR was performed in 96-well plates using 1000 ng of cDNA as template per 20 μL reaction and SYBR^®^ Green Master Mix (England). Thermal cycling comprised 10 min at 95 °C, followed by 40 cycles of 95 °C for 10 s, 58 °C for 10 s, and 72 °C for 20 s. *β-actin* was used as the house-keeping gene. All experiments were repeated three times. Primer sequences are listed in Table 1 [57].

## 5. Conclusions

We synthesized ecofriendly, inexpensive HGRCm-ZnO NPs from hydroponic-cultured ginseng root extract and coated them with an o-carboxymethyl chitosan (CMC) polymer. The biodegradable CMC polymer stabilized the nanoparticles by enhancing the solubility of bioactive ginsenosides besides prompting the biological effects. ZnO NPs were found to have a flower-shaped and crystalline nature by TEM and XRD analyses. At concentrations up to 20 g/mL, an in vitro cytotoxicity investigation showed that HGRCm-ZnO NPs were more toxic to cancerous cells than non-cancerous cells. Additionally, HGRCm-ZnO NPs altered the colony-forming and migration abilities of human lung carcinoma cells (A549). Accordingly, Hoechst and PI staining results revealed that NPs induced apoptosis in cancer cells. However, the cellular apoptosis of cancer cells through the intrinsic mitochondrial pathway was confirmed at the gene expression level by qRT-PCR. Moreover, HGRCm-ZnO NPs increased *BAX* expression and downregulated *Bcl-2* gene expression. Together, these results indicate that HGRCm-ZnO NPs are potentially highly efficacious anti-lung cancer agents.

## Figures and Tables

**Table 1:** Apoptotic primer sequence.

Primer		Sequence
*β-actin*	Forward	5′-CGGGAAATCGTGCGTGAC-3′
	Reverse	5′-AGCTCTTCTCCAGGGAGGA-3′
*BAX*	Forward	5′-AGCAAACTGGTGCTCAAGGC-3′
	Reverse	5′-CCACAAAGATGGTCACTGTC-3′
*Cytochrome c*	Forward	5′-GAGGCAAGCATAAGACTGG-3′
	Reverse	5′-TACTCCATCAGGGTATCCTC-3′
*Bcl-2*	Forward	5′-GTGGTGGAGGAACTCTTCAG-3′
	Reverse	5′-GTTCCACAAAGGCATCCCAG-3′
*Caspase9*	Forward	5′-CGCCACCATCTTCTCCCTG-3′
	Reverse	5′-GCCATGGTCTTTCTGCTCA-3′
*Caspase3*	Forward	5’-CCTCAGAGAGAGACATTCATG-3′
	Reverse	5’-GCAGTAGTCGCCTCTGAAG-3′

## Data Availability

The concern of corresponding author data can be provided.

## Supplementary Materials

The following supporting information can be downloaded at: https://www.mdpi.com/article/10.3390/molecules28020906/s1, Figure S1: Morphology of A549 cells after treatment with different concentrations of HGRCm-ZnO NPs and cisplatin; Table S1: Crystalline size of HGRCm-ZnONPs using Hydroponic Ginseng; Table S2: IC_50_ values of (Cisplatin, CMC, ZnO, HGR-Ex, HGR-ZnO NPs, HGRCm- ZnO NPs) against two cell lines.
