# Supplementary material for: Hydroponic Ginseng ROOT Mediated with CMC Polymer-Coated Zinc Oxide Nanoparticles for Cellular Apoptosis via Downregulation of BCL-2 Gene Expression in A549 Lung Cancer Cell Line
Source: Molecules. 2023 Jan 16;28(2):906. doi: 10.3390/molecules28020906 (PMC9861826; doi:10.3390/molecules28020906)
Supplement: Supplementary file 1 [file molecules-28-00906-s001.zip › Supplementry Figure list.pptx]

## Slide 1
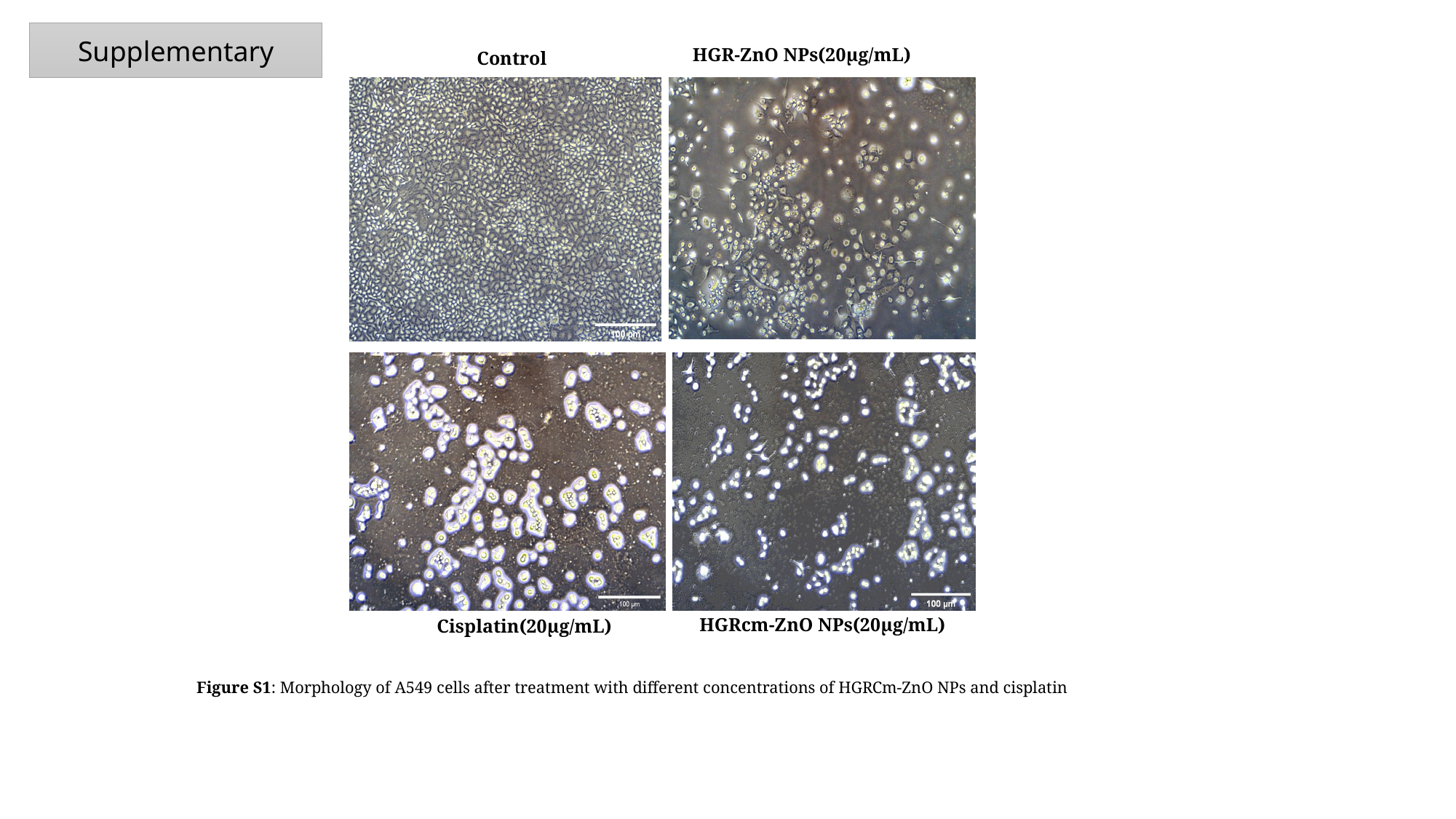

Supplementary
HGR-ZnO NPs(20µg/mL)
Control
HGRcm-ZnO NPs(20µg/mL)
Cisplatin(20µg/mL)
Figure S1: Morphology of A549 cells after treatment with different concentrations of HGRCm-ZnO NPs and cisplatin
