# Supplementary material for: Hydroponic Ginseng ROOT Mediated with CMC Polymer-Coated Zinc Oxide Nanoparticles for Cellular Apoptosis via Downregulation of BCL-2 Gene Expression in A549 Lung Cancer Cell Line
Source: Molecules. 2023 Jan 16;28(2):906. doi: 10.3390/molecules28020906 (PMC9861826; doi:10.3390/molecules28020906)
Supplement: Supplementary file 1 [file molecules-28-00906-s001.zip › Supplementry Table file.docx]

**Table S1.** Crystalline size of HGRCm-ZnONPs using Hydroponic Ginseng.

| **Peak number** | **Peak position angle (2θ** | **D spacing value (A°)** | **FWHM** | **Size (nm)** | **Average size (nm)** |
| --- | --- | --- | --- | --- | --- |
| 100 | 31.82 | 2.80 | .2145 | 28.13 |  |
| 002 | 34.47 | 2.59 | .2234 | 29.47 | 28.87 nm |
| 101 | 36.31 | 2.47 | .2228 | 29.01 |  |
| 102 | 47.59 | 1.90 | .4103 | 16.24 |  |
| 110 | 56.66 | 1.62 | .2436 | 23.56 |  |
| 103 | 62.19 | 1.47 | .4356 | 18.13 |  |
| 200 | 66.46 | 1.40 | .5127 | 19.20 |  |

**Table S2.** IC_50_ values of (Cisplatin, CMC, ZnO, HGR-Ex, HGR-ZnO-NPs, HGRCm- ZnO-NPs) against two cell lines.

| HaCaT (Normal Cell) | IC_50_ with Standard Deviation (µg/mL) | | | | |
| --- | --- | --- | --- | --- | --- |
|  | CMC | ZnO | HGR-Ex | HGR-ZnO-NPs | HGRCm- ZnO-NPs |
|  | 240.19±3.09 | 199.41±3.13 | 121.96±4.19 | 59.42±4.42 | 52.56±2.21 |

| A549  (Lung Cancer Cell) | IC_50_ with Standard Deviation (µg/mL) | | | | | |
| --- | --- | --- | --- | --- | --- | --- |
|  | Cisplatin | CMC | ZnO | HGR-Ex | HGR-ZnO-NPs | HGRCm- ZnO-NPs |
|  | 23.64±3.62 | 55.31±2.77 | 139.11±4.96 | 129.57±2.09 | 39.22±3.04 | 15.74±2.05 |
