# Supplementary material for: Hydroponic Ginseng ROOT Mediated with CMC Polymer-Coated Zinc Oxide Nanoparticles for Cellular Apoptosis via Downregulation of BCL-2 Gene Expression in A549 Lung Cancer Cell Line
Source: Molecules. 2023 Jan 16;28(2):906. doi: 10.3390/molecules28020906 (PMC9861826; doi:10.3390/molecules28020906)
Supplement: Supplementary file 1 [file molecules-28-00906-s001.zip › Supplementry Table file.pdf]

Table S1. Crystalline size of HGRCm-ZnONPs using Hydroponic Ginseng.

**Peak number**	**Peak position angle (2θ**	**D spacing value (A°)**	**FWHM**	**Size (nm)**	**Average size (nm)**
100	31.82	2.80	.2145	28.13	
002	34.47	2.59	.2234	29.47	28.87 nm
101	36.31	2.47	.2228	29.01	
102	47.59	1.90	.4103	16.24	
110	56.66	1.62	.2436	23.56	
103	62.19	1.47	.4356	18.13	
200	66.46	1.40	.5127	19.20	

Table S2. IC_{50} values of (Cisplatin, CMC, ZnO, HGR-Ex, HGR-ZnO-NPs, HGRCm- ZnO-NPs) against two cell lines.

HaCaT (Normal Cell)	IC_{50} with Standard Deviation (μg/mL)				
	CMC	ZnO	HGR-Ex	HGR-ZnO-NPs	HGRCm- ZnO-NPs
	240.19±3.09	199.41±3.13	121.96±4.19	59.42±4.42	52.56±2.21

A549 (Lung Cancer Cell)	IC_{50} with Standard Deviation (μg/mL)					
	Cisplatin	CMC	ZnO	HGR-Ex	HGR-ZnO-NPs	HGRCm- ZnO-NPs
	23.64±3.62	55.31±2.77	139.11±4.96	129.57±2.09	39.22±3.04	15.74±2.05
